# Factors that predict recurrence later than 5 years after initial treatment in operable breast cancer

**DOI:** 10.1186/s12957-016-0988-0

**Published:** 2016-08-24

**Authors:** Pattaraporn Wangchinda, Suthinee Ithimakin

**Affiliations:** Division of Medical Oncology, Department of Internal Medicine, Siriraj Hospital, Mahidol University, Chalermprakiat Building, 13th floor, Siriraj Hospital, 2 Wanglang Rd, Bangkoknoi, Bangkok, 10700 Thailand

**Keywords:** Breast cancer, Clinicopathological, Late recurrence, Luminal breast cancer

## Abstract

**Background:**

Occasionally, breast cancer relapses more than 5 years after initial treatment, sometimes with highly aggressive disease in such late-recurring patients. This study investigated predictors of recurrence after more than 5 years in operable breast cancer.

**Methods:**

We retrospectively analyzed data from patients with recurrent breast cancer treated at Siriraj Hospital. Patients were divided into those whose relapse times were longer or shorter than 5 years. Factors that predicted late recurrence were analyzed in both the overall population and the luminal subgroup. Patterns of relapse, changes in biomarkers, and time to disease progression after first relapse were also recorded.

**Results:**

We included 300 women whose breast cancers recurred between 2005 and 2013, of whom 180 had recurrence within 5 years of diagnosis and 120 later than 5 years (median time to recurrence: 45.43 months; range: 4.4–250.3 months). Tumors larger than 2 cm, lymph node metastasis, and high nuclear grade were related with early recurrence. Estrogen receptor-positive, progesterone receptor-positive, and HER2^−^ disease predicted late recurrence. Almost all late-relapsing patients with luminal tumors had high estrogen receptor (ER^+^) titers (≥50 %) and HER2^−^ disease. Liver and brain were the most common early recurrence sites. Biomarkers did not significantly change by time of recurrence.

**Conclusions:**

ER^+^/PR^+^ and HER2^−^ patients have higher risk of recurrence later than 5 years, especially in patients with high ER titer and low nuclear grade. Larger and node-positive tumors had higher risk of early recurrence.

## Background

Breast cancer, the most common cancer in women, is highly heterogeneous with various clinical courses and outcomes. Adjuvant systemic therapies, including chemotherapy, hormonal treatment, and anti-HER2 (human epidermal receptor) agents have been proven to reduce disease recurrence and prolong survival. Breast cancers are classified into genomically defined subgroups, including four major intrinsic subtypes: luminal A, luminal B, HER2^+^, and triple-negative (TN) tumors. Clinical courses, patterns of metastasis, and prognosis vary among these subgroups. Although most relapses occur during the first 5 years after diagnosis, late recurrence has been reported, especially in luminal breast cancer.

Unlike most solid malignancies, breast cancer may recur 5–10 years after initial treatment. Previous studies reported that high-bulk disease, high proliferative index, and HER2-positive malignancies corresponded to recurrence earlier than 10 years, whereas progesterone receptor-positive (PR^+^) disease was associated with relapse later than 10 years [1, 2]. Follow-up visits are usually scheduled annually for patients who have had five disease-free years or who have completed hormonal treatment. Some patients develop rapid and extensive metastasis during the follow-up intervals; a few of these patients cannot undergo chemotherapy owing to organ dysfunction or lower performance status as a result of widespread metastasis.

Although adjuvant chemotherapy reduces the risk of recurrence in the first 5 years [3], its effect beyond 5 years is unknown. Patients with estrogen receptor-positive (ER^+^) breast cancer benefit from adjuvant tamoxifen, with the greatest benefit in the first 4 years [4] and an additional carry-over reduction of recurrence risk for more than 5 years. Use of tamoxifen extended to 10 years in women with early-stage breast cancer reportedly reduces the risk of late recurrence [5]. Despite the substantial benefit of the 10-year tamoxifen regimen, higher incidence of toxicity, including endometrium cancer and thromboembolism, has also been shown. To improve risks and benefits, patients with high chances of late relapse might be better candidates for such long-term treatment.

The objective of this study was to identify predictors of relapse later than 5 years in early breast cancer. Secondary objectives were identification of predictors of late recurrence specifically in luminal breast cancer, patterns of recurrence, and changes in biomarkers by the time of recurrence. Predicting patients with high chances of late relapse may modify individual follow-up schedules and individualize long-term hormonal treatment in the right patients.

## Methods

All patients who had histologically confirmed breast cancers that recurred after initial treatment were retrospectively reviewed. Patients with incomplete biomarker data were excluded. Data from all patients with recurrent breast cancer were recorded, including demographic, initial stage and tumor biomarkers, date and site of recurrence, any change in biomarkers, and time to progression after first relapse.

Tumors were defined as positive for ER or PR if more than 1 % of cells expressed ER or PR (respectively) [6]. Tumors were considered HER2^+^ if they scored 3+ by immunohistochemistry; or if the ratio of HER2-neu and centromere of 17th chromosome was greater than 2 [7].

For our secondary objectives regarding predictors of late relapse in luminal tumors, we decided to use ER titer as a possible predictive marker. Low ER titer was defined as less than 50 % of tumor cells expressing ER immunohistochemically [8].

All enrolled patients were divided into two groups; those whose relapses were within the first 5 years, and later than 5 years from initial treatment, were respectively grouped as the early recurrence and late recurrence groups. Clinical, pathological, and biomarker data were analyzed for factors that predicted late recurrence in all patients, and in those with luminal tumors. Patterns of relapse and changes of biomarkers over time were also evaluated. The study was approved by Siriraj Institutional Review Board (Protocol number 204/2557(EC1)).

### Statistical analysis

Associations between clinicopathological parameters, immunohistological markers, and late recurrence were assessed using the chi-square and Fisher exact test. Multivariate analysis included only those variables that were positive in univariate analysis and were assessed using multiple binary logistic regression analysis. Also, binary logistic regression was used to evaluate patterns of metastasis in different tumor subtypes. *p* values were two-sided; all confidence intervals were at 95 %. Survival curves were estimated using Kaplan–Meier curves. Analyses were performed using SPSS version 20. This study was approved by the Siriraj Institution Review Board.

## Results

We initially found 554 breast cancer patients who suffered relapses during 2005–2013 using the International Classification of Disease and related health problem 10th revision (ICD10) codes for metastatic breast cancer. After excluding patients with metastatic disease at the first diagnosis, who had incomplete data, or who had refused surgery, 300 patients were eligible for analysis. Their median age was 48 years old. Most of the patients (75 %) were premenopausal at the time of first diagnosis. Their median time to first recurrence was 45.4 months (range: 4.4–250 months). For all patients whose disease had recurred, 78 and 90 % developed recurrence within 10 and 15 years, respectively. Almost all patients received adjuvant systemic treatment and radiotherapy as their physician recommended; only 4 % rejected at least one recommended therapy. Of patients for whom chemotherapy was indicated, 60 % received anthracycline-based chemotherapy and 19 % had combined methotrexate, 5FU, and oral cyclophosphamide. Previously, most patients had received tamoxifen as an adjuvant hormonal therapy.

We divided patients into two groups, early recurrence (within 5 years after diagnosis; *n* = 180; 60 %) and late recurrence (later than 5 years; *n* = 120; 40 %). There was no difference in both early and late recurrence rates among patients who underwent mastectomy and breast-conserving surgery. Among node-positive patients, 70 % were in the early group and 43 % were in the late group (*p* < 0.001). Patients with tumors larger than 2 cm experienced more frequent early recurrence (78 % early group vs 57 % later group). Subtypes of breast cancer also predicted recurrence time. Most of the late group had luminal tumors (85 %) followed by TN tumor (11 %). Among 65 HER2^+^ patients, only 5 (4.2 %) experienced late recurrence, as did only 1.6 % of patients with ER^−^/PR^−^/HER2^+^ tumors (Table 1).

**Table 1:** Patients’ characteristics by time to diagnosis of breast cancer recurrence

		Time to recurrence		Total (*n* = 300)	*p*
		<5 years (*n* = 180)	≥5 years (*n* = 120)		
Mean age, years (SD)		48.45 (10.59)	49.17 (10.61)	300	0.562
Menopausal status	Pre/peri-menopause	138 (76.6)	83 (69.1)	221	0.628
	Postmenopause	42 (23.4)	37 (30.9)	79	
T stage	≤2 cm	40 (22.2)	51 (42.5)	91	<0.001
	>2 cm	140 (77.8)	69 (57.5)	209	
N stage	Node-negative	53 (29.4)	69 (56.6)	122	<0.001
	Node + 1-3 nodes	56 (31.1)	36 (30)	92	
	Node + 4-9 nodes	39 (21.7)	12 (10)	51	
	Node+ ≥ 10 nodes	32 (17.8)	3 (2.5)	35	
N stage	Node-negative	53 (29.4)	69 (56.6)	121	<0.001
	Node-positive	127 (70.6)	51 (43.4)	179	
Grade (*n* = 262)	1	13 (7.6)	19 (20.6)	32	0.002
	2	103 (60.5)	56 (60.8)	159	
	3	54 (31.9)	17 (18.6)	71	
LVI (*n* = 262)	Yes	108 (63.9)	43 (46.2)	151	0.006
	No	61 (36.1)	50 (53.8)	111	
ER	Positive	101 (56.1)	104 (86.6)	205	0.001
	Negative	79 (43.9)	16 (13.4)	95	
PR	Positive	76 (42.2)	95 (79.1)	171	0.001
	Negative	104 (57.8)	25 (20.9)	129	
HER2	Positive	60 (33.3)	5 (4.1)	65	0.001
	Negative	120 (66.7)	115 (95.9)	235	
Subtypes	Luminal A/B/HER2^−^	76 (42.2)	102 (85.0)	178	0.001
	Triple-negative	44 (24.4)	13 (10.8)	57	
	HER2	35 (19.4)	2 (1.6)	37	
	ER^+^ or PR^+^ and HER2^+^	25 (14.0)	3 (2.6)	28	
Surgery (*n* = 293)	Mastectomy	163 (90.5)	106 (88.3)	269	0.31
	Breast-conserving	12 (6.7)	12 (10)	24	
Adjuvant systemic treatment	None	8 (4.4)	4 (3.3)	12	0.001
	Chemotherapy	81 (45.0)	20 (16.6)	101	
	Endocrine therapy	17 (9.4)	35 (29.1)	52	
	Chemo-endocrine	74 (41.2)	61 (51.0)	135	
Adjuvant RT	Yes	108 (60.0)	47 (39.1)	155	0.073

Most patients with tumors larger than 2 cm or lymph node metastasis relapsed early, while higher proportion of patients with small tumors or negative lymph nodes were found among patients who experienced late recurrence (Table 2). In multivariate analysis, factors that predicted late recurrence were ER^+^, PR^+^ status, and HER2^−^status, whereas HER2^+^ status predicted early relapse (Table 2).

**Table 2:** Univariate and multivariate analyses of factors correlated with late recurrence (≥5 years)

Factors		Univariate analysis		Multivariate analysis	
		RR/CI	*p*	RR/CI	*p*
T stage	≤2 cm	2.59/1.57–4.28	<0.001	2.09/1.09–3.99	0.026
	>2 cm	1		1	
N stage	−	3.24/2.0–5.26	0.137		
	+	1			
Grade	1	1		1	
	2	0.39/0.18–0.85	0.017	0.39/0.15–0.97	0.043
	3	0.24/0.1–0.58	0.001	0.46/0.17–1.3	0.142
LVI	+	0.49/0.29–0.81	0.006	0.82/0.44–1.55	0.546
	−	1		1	
ER	+	5.08/2.78–9.29	<0.001	3.25/1.1–9.6	0.033
	−	1			
PR	+	5.20/3.06–8.84	<0.001	1.86/0.75–4.62	0.184
	−	1		1	
HER2	+	0.09/2.35–29.3	<0.001	0.12/0.03–0.43	0.001
	−	1		1	

*RR* relative risk, *CI* 95 % confidence interval, *LVI* lymphovascular invasion

Because luminal tumors had a substantial chance of late recurrence, we investigated factors that predict late relapse in this specific population. Among the enrolled 300 patients, 206 (68.7 %) had ER^+^ and/or PR^+^ tumors. Of 206 patients with luminal tumor, 105 (50.9 %) experienced late recurrence; as with the overall population, early recurrence occurred in luminal tumor patients with larger tumor, positive lymph nodes, and *HER2* amplification (Tables 3 and 4). Two out of 74 late-relapse patients (2.8 %) had low ER titers, and 15 out of 92 patients with early relapse (18 %) had low ER (*p* = 0.002). Patients with low ER titers or whose tumors were ER^−^ or PR^−^ had significantly less late relapse (Table 3). Multivariate analysis associated highly positive ER intensity with late recurrence (Table 4).

**Table 3:** Patient characteristics by time to diagnosis of recurrence of breast cancer in luminal subgroup

		Time to recurrence		Total (*n* = 206)	*p*
		<5 years (*n* = 101)	≥5 years (*n* = 105)		
Mean age, years (SD)		49.17 (10.5)	48.45 (10.6)	206	0.562
Menopausal status	Pre/peri-menopause	74 (73.2)	71 (67.6)	145	0.377
	Postmenopause	27 (26.8)	34 (32.4)	61	
T stage	≤2 cm	19 (18.9)	42 (40.0)	61	0.001
	>2 cm	82 (81.1)	63 (60.0)	145	
N stage	Node-negative	29 (28.7)	59 (56.2)	88	<0.001
	Node-positive	72 (71.3)	46 (43.8)	118	
Grade (*n* = 174)	1	9 (9.4)	15 (18.9)	24	0.019
	2	59 (62.1)	51 (64.5)	110	
	3	27 (28.5)	13 (16.6)	40	
Lymphovascular invasion (*n* = 176)	Yes	59 (62.1)	42 (51.8)	101	0.172
	No	36 (37.9)	39 (48.2)	75	
ER titer (*n* = 156)	High (≥50 %)	67 (81.7)	72 (97.2)	139	0.002
	Low (<50 %)	15 (18.3)	2 (2.8)	17	
ER^−^ or PR^−^		26 (25.7)	4 (3.8)	30	<0.001
HER2^+^		25 (24.7)	3 (2.8)	28	<0.001
Adjuvant therapy	None	4 (3.9)	2 (1.9)	6	0.047
	Chemotherapy	7 (6.9)	7 (6.6)	14	
	Endocrine therapy	17 (16.8)	35 (33.3)	52	
	Chemo-endocrine	73 (72.4)	61 (58.2)	154

**Table 4:** Univariate and multivariate analyses of recurrence later than 5 years in patients with luminal tumors

Factors	Category	Later than 5 years	Univariate analysis		Multivariate analysis	
		/total *n*	RR/CI	*p*	RR/CI	*p*
T stage	≤2 cm	45/65	3.04/1.63–5.67	<0.001	3.96/1.66–9.43	0.002
	>2 cm	60/141	1		1	
N stage	Node^−^	60/89	3.31/1.86–5.91	<0.001	3.47/1.51–7.99	0.003
	Node^+^	45/117	1		1	
Grade	1	15/23	1		1	
	2	53/115	0.46/0.18–1.16	0.099	0.15/0.04–0.62	0.008
	3	14/41	0.28/0.10–0.81	0.019	0.23/0.05–1.05	0.057
ER titer	High ≥ 50 %	72/139	8.06/1.78–36.57	0.007	10.4/1.88–57.5	0.007
	Low < 50 %	2/17	1		1	
ER^−^ or PR^−^	Yes	101/176	8.75/2.93–26.15	<0.001	2.95/0.12–74.6	0.511
	No	4/30	1		1	
HER2	Positive	3/28	0.09/0.03–0.31	<0.001	0.44/0.01–16.9	0.655
	Negative	102/178	1		1	

*RR* relative risk, *CI* 95 % confidence interval

**Fig. 1:**
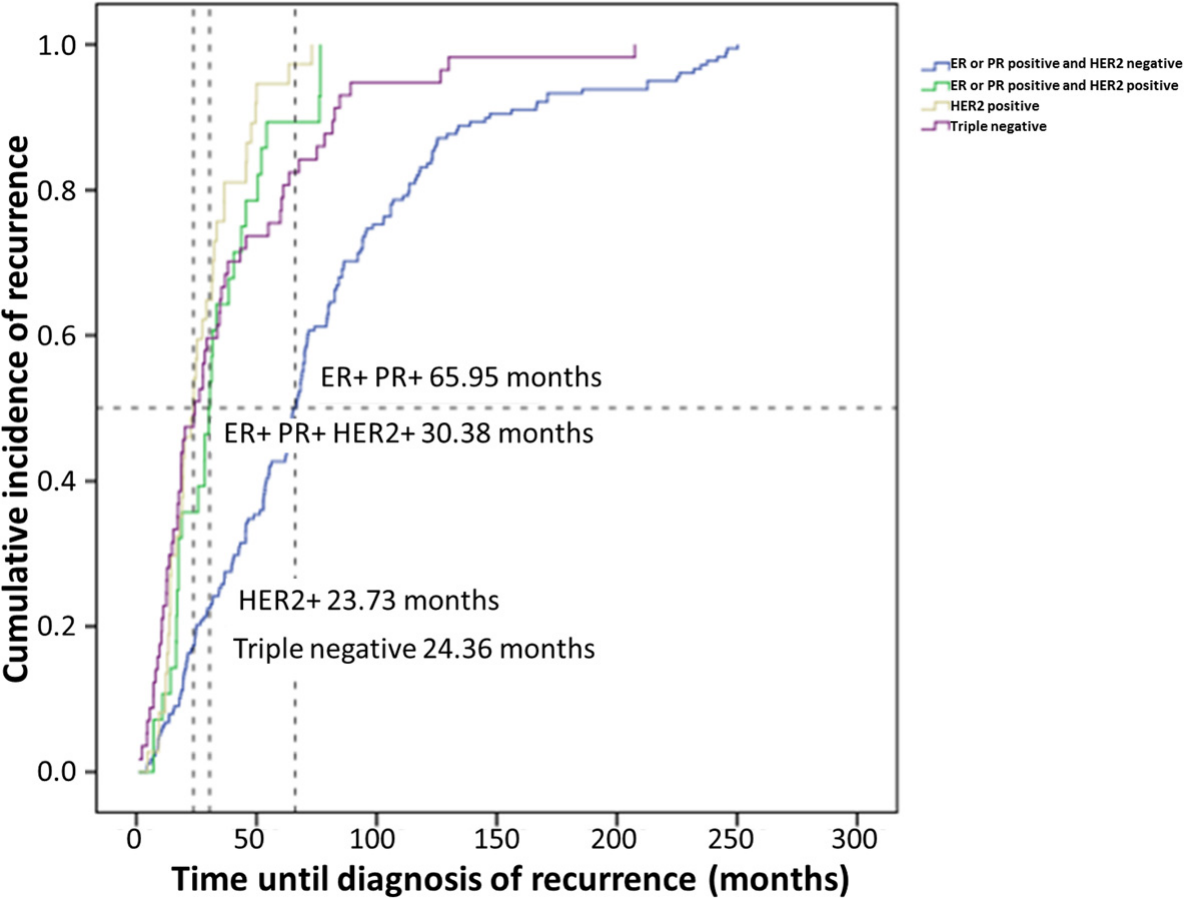
Cumulative incidence of first relapse of breast cancer by breast cancer subtype

The different breast cancer subtypes showed different times to disease progression. As expected, the luminal/HER2^−^ subgroup had the longest disease-free survival (DFS; median DFS: 65.95 months), whereas patients with TN or ER^−^/HER2^+^ disease had worse prognoses (median DFS: 23.7 and 24.4 months, respectively; Fig. 1). However, at that time, 92.2 % of HER2^+^ patients received no adjuvant trastuzumab owing to reimbursement issues and financial constraints.

**Fig. 2:**
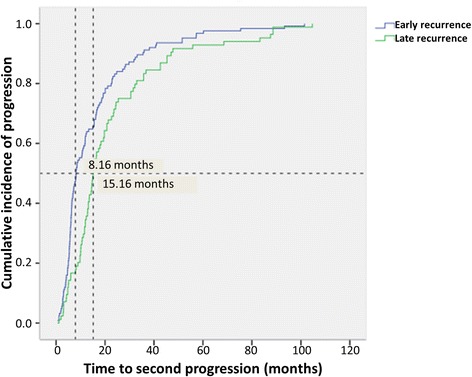
Progression-free survival after first relapse categorized by early or late recurrence (months)

Liver and brain were the most common metastasis sites for patients with early relapse. However, lung or soft tissue and bone metastasis occurred throughout the follow-up period. Patients with locoregional recurrence as first relapse site experienced longer DFS (median: 82.55 months; range: 7.17–234.9 months) compared with those with distant metastasis (median DFS: 42.58 months; range: 4.40–250.27 months). In the overall population, metastasis to the central nervous system was largely found in patients with TN and HER2^+^ cancers. Luminal HER2^−^ tumors were predominantly metastasized to the bone and were less likely to spread to the liver at first relapse (Tables 5 and 6). Patterns of relapse did not significantly differ between luminal patients who relapsed late or early (Table 7). The late-relapse group had longer median time to disease progression (15.16 months) than did the early group (8.16 months; *p* = 0.008) (Fig. 2).

**Table 5:** Metastasis patterns according to the time to diagnosis of breast cancer recurrence

Site of recurrences	Time to recurrence		Total (*n* = 300)	*p*
	<5 years (*n* = 180)	≥5 years (*n* = 120)		
Pulmonary metastasis	132 (73.3)	91 (75.8)	223	0.102
Bone metastasis	82 (45.5)	58 (48)	140	1.051
Brain metastasis	22 (12.2)	4 (3.3)	26	0.007
Liver metastasis	57 (31.6)	20 (16.6)	77	0.004
LN metastasis	38 (21.1)	29 (24.1)	67	0.534
Skin metastasis	32 (17.7)	22 (18.3)	54	0.902
Locoregional recurrence	10 (5.0)	16 (8.0)	26	0.019
Distant metastasis	170 (94.4)	104 (86.6)	274	0.088
Multiple site metastasis	132 (73.3)	76 (63.3)	208	0.066

**Table 6:** Relative risk for site of first recurrence by breast cancer subtype in 300 women

Site	TNBC (*n* = 57)		HER2 (*n* = 37)		ER/PR/HER2 (*n* = 28)		ER/PR (*n* = 178)	
	RR/CI	*p*	RR/CI	*p*	RR/CI	*p*	RR/CI	*p*
Lung	0.959/0.49–1.84	0.901	0.655/0.36–1.19	0.166	0.495/0.22–1.11	0.083	1.399/0.82–2.35	0.207
Bone	0.554/0.30–1.00	0.052	0.708/0.40–0.23	0.223	1.159/0.53–2.52	0.710	1.849/1.15–2.95	0.010
Brain	2.493/1.04–5.92	0.034	2.489/1.07–5.78	0.030	3.436/1.25–9.44	0.024	0.222/0.09–0.54	<0.001
Liver	1.439/0.76–2.70	0.256	4.897/2.71–8.82	<0.001	1.70/0.74–3.86	0.201	0.220/0.12–0.38	<0.001
LN	1.641/0.85–3.13	0.131	2.160/1.17–3.96	0.012	1.748/0.75–4.06	0.191	0.397/0.22–0.69	0.001
Skin	1.112/0.53–2.32	0.777	0.577/0.25–1.29	0.177	0.740/0.24–2.22	0.551	1.327/0.71–2.45	0.365

*CI* 95 % confidence interval, *RR* relative risk, *TNBC* triple-negative breast cancer

**Table 7:** Metastasis patterns in patients with luminal subtypes according to the time to diagnosis of breast cancer recurrence

Site of first recurrence	Time to recurrence		Total (*n* = 206)	*p*
	<5 years (*n* = 101)	≥5 years (*n* = 105)		
Pulmonary metastasis	71 (70.2)	83 (79.0)	154	0.150
Bone metastasis	58 (57.4)	50 (47.6)	108	0.160
Brain metastasis	9 (8.9)	4 (3.8)	13	0.134
Liver metastasis	20 (19.8)	15 (14.2)	35	0.294
LN metastasis	13 (12.8)	24 (22.8)	37	0.062
Skin metastasis	20 (19.8)	19 (18.0)	39	0.756

Eighty of the 300 patients were re-biopsied at the time of recurrence. Most (79 %) had biomarkers similar to those seen at their initial diagnoses. Discordant biomarkers were infrequent and did not significantly differ by relapse time (Table 8).

**Table 8:** Discordance of hormonal receptor and HER2 status between primary and metastatic samples (only in patients with rebiopsies and with diagnoses confirmed by pathologist; *n* = 80)

		Change of HR and/or HER2 status	
		Early recurrence (*n* = 40)	Late recurrence (*n* = 40)
No change ER status		36	36
ER change	Pos → neg	2	2
	Neg → pos	2	2
No change PR status		35	37
PR change	Pos → neg	2	2
	Neg → pos	3	1
No change HER2 status		35	38
HER2 change	Pos → neg	1	0
	Neg → pos	4	2

Changes were seen in 17 patients (some patients had more than 1 biomarker changed)

## Discussion

As with most solid cancers, breast cancer relapse rates are highest in the first 5 years following initial treatment and decline to a steady state after 7–8 years. Late relapse of breast cancer is occasionally found after DFS of 5–10 years. Breast cancer patients who had high disease bulk (such as large tumors or positive lymph nodes) generally had higher chances of both early and late recurrence [1]. A previous study showed that recurrence that occurred later than 10 years was associated with positive lymph node or PR^+^ disease [2].

Our study confirmed that tumor size and regional lymph node involvement were predictive of early recurrence. In addition, biomarkers were stronger predictors of early or late recurrence. HER2^+^ status predicted recurrence within 5 years; only five HER2^+^ patients (4 %) were in the late group, and three of these five also had positive hormone receptors. Because TN breast cancer is known to be an especially aggressive subtype, our study showed cancers of this subtype made up 10 % of the late group, compared with 24 % of the early group. These patterns have implications for follow-up scheduling in patients whose DFS has been longer than 5 years; HER2^+^ patients can be set for annual visits, whereas ER^+^ patients (especially those who had high ER titer) might need more frequent follow-up visits because the risk of recurrence is still retained.

Patients with hormone receptor-positive breast cancer remain at risk of recurrence for as long as they survive. For that reason, many investigators have explored the benefit of extended therapy beyond the standard treatment of 5 years. Recently, several studies showed that extended tamoxifen reduces late recurrence risk by one third compared with no further hormonal therapy beyond 5 years [5, 9]. Extended use of aromatase inhibitors has also been shown to reduce the risk of late relapse by nearly 50 % compared with no further treatment [10, 11]. However, prolonged endocrine therapy leads to adverse events (such as hot flashes, sexual dysfunction, uterine bleeding, or osteoporosis) in approximately half of patients who take it. Therefore, treating only patients with a clear chance of recurrence but not those unlikely to develop late recurrence is a reasonable practice. The question is: Which patient has risk of late relapse and will benefit from extended hormonal therapy?

We therefore explored more patients with luminal tumors. Identifying patients with high risk of late relapse and who may benefit from extended hormonal therapy for up to 10 years could aid in treatment decisions. In luminal tumors, titers of ER and HER2 predicted late relapse. Patients with ER titers <50 % or concomitant HER2^+^ disease experienced significantly lower rates of late recurrence. Therefore, benefits of extended hormonal treatment in these patients might not outweigh the risk of treatment. Given that coexpression of ER and PR were related to late recurrence compared with patients who had only ER or PR, our findings support greater benefit of extended letrozole treatment in patients who had ER^+^/PR^+^ tumors, as shown in subsequent analysis of MA17 [12].

Recently, studies using multi-parameter assays, for example the 12-gene EndoPredict, Breast Cancer Index (BCI), or 50 gene *PAM50 ROR*, investigated the risk of late recurrence [13, 14]. These molecular assays can separate patients with low risk of developing recurrence at 5–10 years or 10–15 years [15]. Among these parameters, *PAM50 ROR* was the strongest molecular prognostic factor for late recurrence and possibly predicted who could benefit most from extended hormonal treatment [1]. However, these data were exploratory, and validation of their applicability to specific subgroups is needed. These multi-parameter assays are also limited by availability and cost. Clinicopathological parameters remain the key to clinical decision-making in our practice.

As for patterns of metastasis, patients who relapsed early tended to have liver and brain metastasis, whereas metastasis to the bone occurred independently of the period of time to relapse. For the course of disease, Fig. 2 shows slower disease progression in the late group. These can be explained by more indolent biology in patients with late relapse.

The main limitation of our study was its retrospective design based on clinicopathological data. Some past adjuvant therapies also do not represent current standard practices; for instance, trastuzumab was rarely used in high-risk HER2^+^ breast cancer patients because of availability and reimbursement issues. In addition, some parameters such as Ki67 have not been used generally at the time of breast cancer diagnosis in our institution.

## Conclusions

Larger and node-positive tumors associate with greater chances of early recurrence. Factors that predict late recurrence are luminal tumors, especially with concomitant PR-positive status, high ER titer, small tumor, negative lymph node, low grade, and HER2^−^ disease.

## Abbreviations

ER: Estrogen receptor
HER2: Human epidermal receptor 2
TN: Triple-negative
PR: Progesterone receptor
ICD10: International classification of disease and related health problem 10th revision
DFS: Disease-free survival
